# Pediatric Enteroviral Central Nervous System Infections in Bialystok, Poland: Epidemiology, Viral Types, and Drivers of Seasonal Variation

**DOI:** 10.3390/v12080893

**Published:** 2020-08-15

**Authors:** Kacper Toczylowski, Magdalena Wieczorek, Ewa Bojkiewicz, Magdalena Wietlicka-Piszcz, Beata Gad, Artur Sulik

**Affiliations:** 1 Department of Pediatric Infectious Diseases, Medical University of Bialystok, Waszyngtona 17, 15-274 Bialystok, Poland; ewa.bojkiewicz@umb.edu.pl (E.B.); artur.sulik@umb.edu.pl (A.S.); 2 Department of Virology, National Institute of Public Health—National Institute of Hygiene, Chocimska 24, 00-791 Warsaw, Poland; mrechnio@pzh.gov.pl (M.W.); bgad@pzh.gov.pl (B.G.); 3 Department of Theoretical Foundations of Biomedical Sciences and Medical Computer Science, Nicolaus Copernicus University in Torun, L. Rydygier Collegium Medicum in Bydgoszcz, 9 M. Skłodowska-Curie St., 85-094 Bydgoszcz, Poland; mpiszcz@cm.umk.pl

**Keywords:** enterovirus, aseptic meningitis, seasonality, epidemiology, phylogenetic analysis, Poland

## Abstract

Enteroviruses are common causes of infections of the central nervous system (CNS) that in temperate climates tend to peak in the summer. The aim of the study was to describe epidemiology, drivers of seasonality, and types of enteroviruses causing infections of the CNS in children in Northeastern Poland. We prospectively collected data on children hospitalized with infection of the CNS attributed to enteroviruses in Bialystok, Poland, from January 2015 to December 2019. In total, 224 children were included. Nineteen different enterovirus types were identified in isolates collected from 188 children. Coxsackie B5 (32%), echovirus 30 (20%), and echovirus 6 (14%) were the three most common types. Enteroviruses were more prevalent during the summer–fall season. Infections caused by echovirus 30 peaked early in June and coxsackievirus B5 in July, whereas echovirus 6 peaked late in October. Phylogenetic analyses of these three enterovirus types showed multiple lineages co-circulating in this region. Mean air temperatures and precipitation rates were independently associated with monthly number of cases. Considering lack of effective treatment or vaccine, easy transmission of enteroviruses between susceptible individuals, their high mutation rate and prolonged time of viral shedding, continued monitoring and surveillance are imperative to recognize enteroviral infections of the CNS and the changes in circulation of enteroviruses in Poland.

## 1. Introduction

Enteroviruses (EVs) belong to the *Picornaviridae* family and originally were classified into four groups: polioviruses, coxsackievirus A, coxsackievirus B, and echoviruses. According to the current taxonomy based on virus genomic and biological properties, EVs are divided into 15 species, of which EV species A-D and Rhinovirus species A-C infect humans [1]. Worldwide, enteroviruses are responsible for nearly a billion infections in people annually [2]. Clinical syndromes include febrile rash, hand-foot-and-mouth disease, acute respiratory syndrome, and infections of the central nervous system (CNS). Enteroviral disease is reported year-round but exhibits a peak in the summer season. The observed seasonality can be partially explained by climatic factors, but the drivers of temporal patterns remain largely unknown [3].

Enteroviruses, specifically polioviruses, have a well-defined pathogenicity in the human CNS. Due to the success of the poliovirus eradication program, there has been near global eradication of clinical poliomyelitis [4]. Infections of the CNS caused by non-polio EVs generally have a good prognosis, but the outcome is determined by the type of the enterovirus causing the infection. Non-polio EVs are monitored within the framework of the global WHO polio surveillance network [5]. Enterovirus A71, echovirus 13, and echovirus 11 were recently reported to be the most common enterovirus types associated with acute flaccid myelitis cases similar to clinical poliomyelitis [6]. Continuous surveillance of EV circulation shows that different long-term circulation patterns for individual types exist. For example, surveillance in the USA recorded large and periodic outbreaks of echovirus 9, whilst coxsackievirus B4 and other types had endemic patterns with relatively stable circulation [2]. Monitoring EV circulation is important because changes in predominant serotypes can be accompanied by large-scale outbreaks or increase in cases of severe neurological disease [7].

A large outbreak of enteroviral meningitis caused by echovirus 30 was noted in Northeastern Poland in 2014 with nearly 300 children hospitalized. The outbreak caused a 35-fold increase in hospitalization rates in comparison to the previous year [8]. Acute flaccid paralysis associated with enterovirus 71 was also previously reported in Poland [9]. Recently we have shown that enteroviruses are the most frequently detected pathogens in children with infection of the CNS hospitalized in Bialystok, Poland [10]. To date, however, data on epidemiology of enteroviral CNS disease in Poland are limited. The objective of the study was to characterize molecular epidemiology of enterovirus strains in children with meningitis and encephalitis in Poland. Additionally, to give insights into drivers of seasonality of enteroviruses, we analyzed relationships between climatic factors and numbers of hospitalized children.

## 2. Materials and Methods

### 2.1. Study Design and Participants

In this single-center, prospective, observational cohort study, children were recruited from the Medical University of Bialystok Children’s Clinical Hospital in Poland between January 2015 and December 2019. Bialystok is located in Northeastern Poland. The hospital provides care for all children with infections of the CNS in the region, which is inhabited by approximately 1.2 million people, including 206,000 children [11].

Meningitis was defined as the presence of symptoms consistent with meningitis and cerebrospinal fluid pleocytosis (CSF) (5> cells per µL) [12]. Encephalitis was defined according to the International Encephalitis Consortium [13] as altered mental status (defined as decreased or altered level of consciousness, including change in personality, lethargy) for over 24 h with no alternative cause identified and two of the following: seizures, focal neurologic findings, EEG (electroencephalography) or MRI (magnetic resonance imaging) abnormalities suggestive of encephalitis, CSF pleocytosis and fever.

Patients were eligible for study inclusion if they were younger than 18 years, had clinically diagnosed meningitis or encephalitis, and had an enterovirus detected in CSF or stool specimens. Children diagnosed with a CNS infection attributed to other or unknown pathogens were not included in the study. Detection of EVs was made using the method described below.

### 2.2. EV Molecular Diagnosis

All collected CSF samples were tested with the diagnostic pan-enterovirus RT-PCR (EV PCR) or, from August 2019 on, with the Xpert EV (Cepheid, Sunnyvale, CA, USA). All collected stool samples were tested with the EV PCR. The Xpert EV assay was done according to the manufacturer’s instructions. The EV PCR method involved viral RNA extraction from 140 μL of the sample using spin columns (Qiagen, Venlo, Netherlands) following the manufacturer’s instructions. RT-PCR was carried out based on the WHO manual [14], using pan-enterovirus primers specific for 5′UTR region, which is highly conserved among all human enteroviruses. Amplification products were analyzed in 2% agarose gels, GelRed-stained, and examined under a UV DNA trans-illuminator.

Stool and CSF samples collected during the hospital stay were stored at −80 °C before viral typing.

### 2.3. Virus Isolation in Cells

Stool samples, both positive and negative in EV PCR and positive in EV PCR CSF samples (if available), were cultured for virus isolation using human rhabdomyosarcoma (RD) and a mouse cell line carrying the poliovirus receptor (L20B). For organizational reasons, CSF samples that were positive in Xpert EV were not cultured, but stool samples collected from those patients were used instead. Stool samples were processed according to the standard procedure recommended by WHO [14]. RD and L20B cells were cultivated in minimal essential medium (MEM) supplemented with 10% fetal bovine serum. A volume of 200 μL of sample was inoculated into tubes with RD and L20B cells. The tubes were incubated at 36 °C and were examined daily. After 5 days, the tubes were frozen and thawed and re-passaged, and another 5-day examination was performed. Each specimen underwent two passages in RD and L20B cells. All detected enteroviruses were identified by sequencing.

### 2.4. Enterovirus Identification

To identify the enterovirus type in positive cell cultures, RT-PCR specific for a sequence of the viral protein 1 (VP1) region for species A and B was performed. Viral RNA was extracted from NPEV-positive cell culture supernatant using QIAamp Viral RNA Mini Kit (Qiagen) following the manufacturer’s instructions. The complete VP1 coding region for species B and 684 base pair region of VP1 gene for species A was amplified with nested reverse transcription PCRs using Superscript III (Invitrogen, Waltham, MA, USA), specific primers and PCR cycling times and temperature as previously described [15]. Amplified products were analyzed in 1.5% agarose gels, GelRed-stained, and examined under a UV DNA transilluminator. The resulting DNA templates were processed in a cycle sequencing reaction with a BigDye 3.1 according to manufacturer’s protocol using inner primers to read the sequence between positions 2385–3016 for species A and 2392–3477 for species B. The product of sequencing reaction was run in an automated genetic analyzer (Applied Biosystems, Waltham, MA, USA).

### 2.5. Sequence Analysis

The resulting sequences were manually edited using the BioEdit program and examined in terms of the closest homologue sequence using BLAST software. The sequences of isolated strains were aligned with the reference strains. The complete capsid sequences of the three most frequently detected enterovirus types were analyzed. A phylogenetic tree was computed using the neighbor-joining method with bootstrap 1000 replicates. Molecular and phylogenetic analyses were conducted using MEGA version 10.0.5 [16]. Sequences have been assigned GenBank accession numbers MT347641-MT347677, MT350720-MT350780, MT385498-MT385502, MT385504-MT385525.

### 2.6. Meteorological Data

Daily mean temperature, relative humidity, precipitation rate and duration, sunshine duration and wind speed data in Bialystok for the same period were obtained from the Institute of Meteorology and Water Management—National Research Institute (Warsaw, Poland).

### 2.7. Statistical Analysis

The summary statistics for continuous variables are presented as a median with interquartile range (IQR), categorical variables are presented as frequencies. Differences between groups were analyzed by the Wilcoxon or Kruskal-Wallis test. To assess the strength of the potential association between the incidence of enteroviral infections of the CNS and the environmental factors, the Pearson’s correlation coefficient was calculated. The association between the incidence of infections and the meteorological factors has also been studied by the generalized linear mixed effects model (GLMM) with the Poisson distribution. GLMM is a class of models that enable the modeling of longitudinal non-normal data of many kinds of response variables. The Poisson distribution is used for modeling of continuous variables—in our case the number of EV cases. The meteorological factors have been included in the model as covariates and the number of EV cases (*Y*) as the dependent variable. The year of observation was included in the model as the random effects term (*u*) in order to account for potential variation between years. The results of the estimation of the model are reported as relative risk (risk ratio) (RR) with 95% confidence intervals. Relative risk is the ratio of the probability of the outcome (probability of the EV infection) in the group exposed to the change in a climatic factor to the probability of the outcome in an unexposed group.

First, the associations between the number of EV cases and the considered environmental factors was modelled for each meteorological factor separately (univariable models). Subsequently the model with multiple factors was created (multivariable model), where, initially, all considered meteorological factors (temperature (*Temp*), relative humidity (*Hum*), wind speed (*Wind*), precipitation (*Prec*), sunshine duration (*Sun*) and precipitation duration (*PrecD))* were included in the model as covariates:

logY=Temp + Prec + Sun + Hum + Wind + Prec + Sun + PrecD + u

Then, to select meteorological factors independently associated with the incidence of infections, the backward elimination feature selection procedure was applied, and the non-significant climatic factors were skipped from the model. The results were considered statistically significant when the p-value was less than 0.05. The statistical analysis was performed with the use of TIBCO Software Inc. (2017) Statistica, version 13 (Palo Alto, CA, USA), and the R-software, version 3.6.2, packages lme4, gls and r2glm (The R Foundation for Statistical Computing, Vienna, Austria).

### 2.8. Ethical Considerations

The study was conducted in accordance with the Guidelines for Good Clinical Practice. Ethical approval was given by The Bioethical Commission of The Medical University of Bialystok (decisions no. APK.002.186.2020, approved 30-04-2020, and R-I-002/260/2015 approved 06-01-2015). Written informed consent was obtained from parents or carers.

## 3. Results

### 3.1. Virus Isolation and Typing

**Figure 1:**
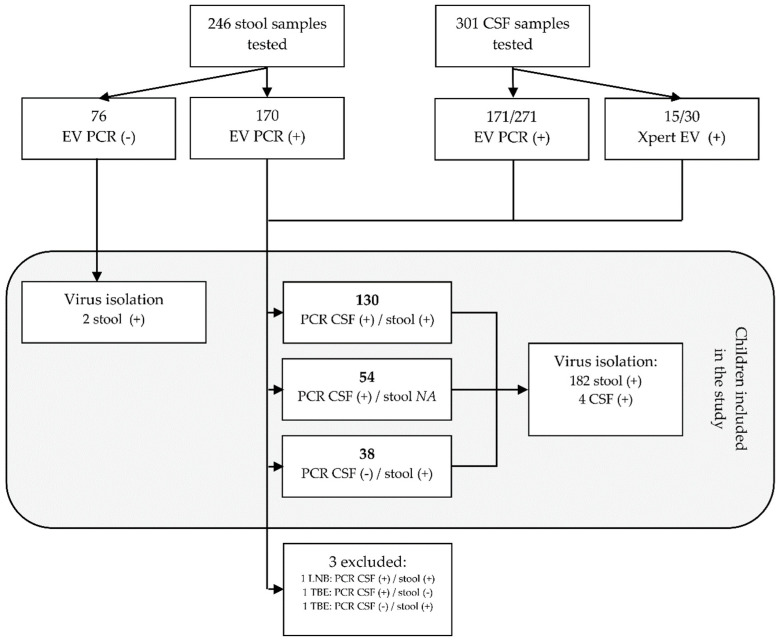
The flowchart representing the process of selecting children for the study. A total of 345 children with signs of meningitis of encephalitis were tested for enteroviruses. Of those, 224 were diagnosed with central nervous system (CNS) infection caused by enteroviruses. Abbreviations: EV PCR, diagnostic pan-enterovirus RT-PCR; virus isolation, virus isolation in cell cultures; CSF, cerebrospinal fluid; LNB, Lyme neuroborreliosis; TBE, tick-borne encephalitis; NA, not available for EV PCR.

Between 1 January 2015 and 31 December 2019, 246 stool and 301 CSF samples collected from 345 children with meningitis or encephalitis presenting to the Medical University of Bialystok Children’s Clinical Hospital were tested for enteroviruses. A total of 186 (62%) CSF and 170 (69%) stool samples were positive. That includes 171 (63%) of 271 CSF samples that were positive in EV PCR and 15 (50%) of 30 positive in Xpert EV (Figure 1). Based on the detection of EVs in those samples, a total of 224 children were diagnosed with CNS infection associated with enteroviruses. The remaining 121 children were diagnosed with tick-borne encephalitis, Lyme neuroborreliosis, or infection of the CNS caused by other or unknown pathogens. In 184 (82%) of children with enteroviral CNS disease, the diagnosis was made after detecting enteroviruses in CSF samples. That includes 130 (58%) children with both the CSF and stool samples positive, and 54 (24%) children with positive CSF only, in whom stool samples were not tested with EV PCR. In 38 (17%) the CSF was negative, but EVs were detected by EV PCR in stool samples. In 2 (1%) children the diagnosis was made after the detection of enteroviruses in the cell culture isolation only. Two stool samples and two CSF samples collected from three children were positive in EV PCR, but those children were finally excluded from the analysis as they were diagnosed with tick-borne encephalitis or Lyme neuroborreliosis.

Virus isolation in cell cultures was done in 261 stool samples and in 13 CSF samples. Of those 261 samples, 76 (29%) stool samples were negative in EV PCR, 170 (65%) were positive in EV PCR, and 15 (6%) samples were collected from children who tested positive in Xpert EV. All 13 CSF samples tested positive in EV PCR. Virus isolation was successful in 184 (70%) stool samples, including 167 with positive detection of EVs in EV PCR, 2 with negative EV PCR, and 15 collected from children diagnosed with Xpert EV. Enteroviruses were isolated in 4 (30%) CSF samples only.

Considering children diagnosed with enteroviral infection of the CNS only, the isolation was done in 195 (87%) children and was successful in 188 (96%) of them. Cell culture isolation was not done in samples collected from 29 (13%) children and failed in 7 (3%) children. Of those 7, virus isolation failed in six CSF samples and in one stool sample. Virus typing was successful for all positive cell cultures. Overall, a total of 19 different types of enteroviruses were detected (Table 1). The majority of identified enteroviruses belonged to the enterovirus group B (EV-B) with 178 (95%) viruses distributed among 15 types. Ten viruses (5%) belonged to 4 types within the group A (EV-A).

**Figure 2:**
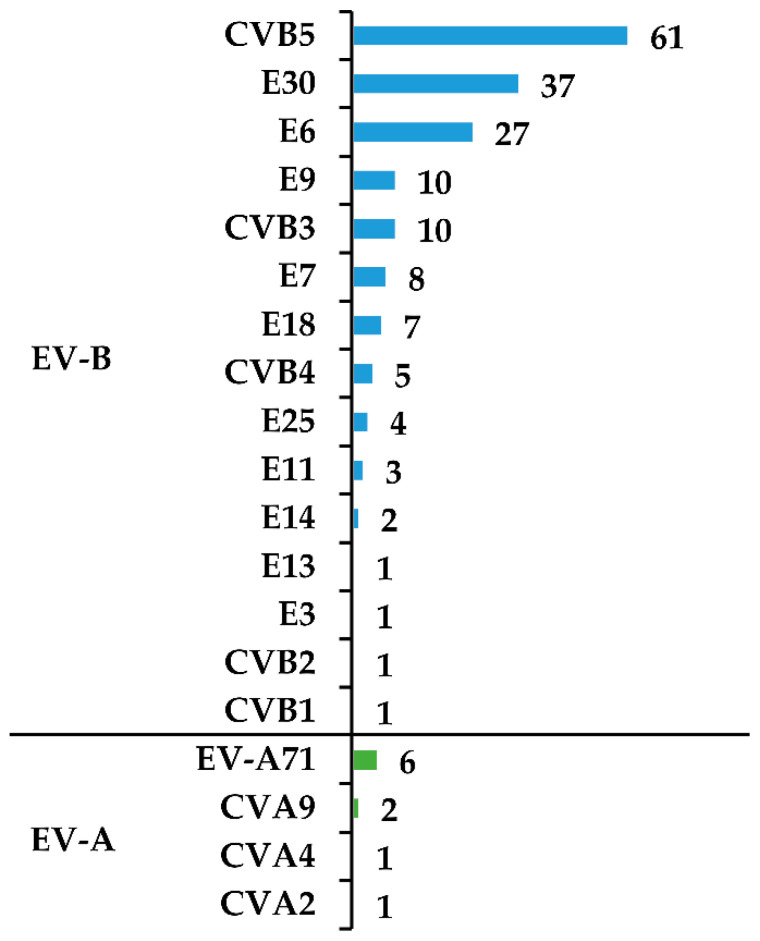
Enterovirus types detected in children with infection of the CNS attributed to enteroviruses. Abbreviations: EV-A, enterovirus A species; EV-B, enterovirus B species; CV, coxsackievirus; E, echovirus.

Coxsackievirus B5 (CVB5) was the most commonly identified enterovirus type detected in 61 (32%) samples, followed by echovirus 30 (E30) detected in 37 (20%) cases, and echovirus 6 (E6) in 27 (14%) cases. Enterovirus A71 (EV-A71) was detected in 6 children (Figure 2).

### 3.2. Clinical Features of the Study Population

Clinical features are shown in Table 2. The median age of hospitalized children was 8.1 years (range 4 months–17 years). Ninety-five (42%) children were under 7, and 89 (40%) between 7 and 14 years old. One hundred-forty-seven (66%) children were boys. The male-to-female ratio in the entire cohort was 1.91. There were, however, slight differences in the male-to-female ratio between children infected with CVB5, E30, E6, and other EVs (1.90, 1.64, 1.25, 2.30, respectively).

**Figure 3:**
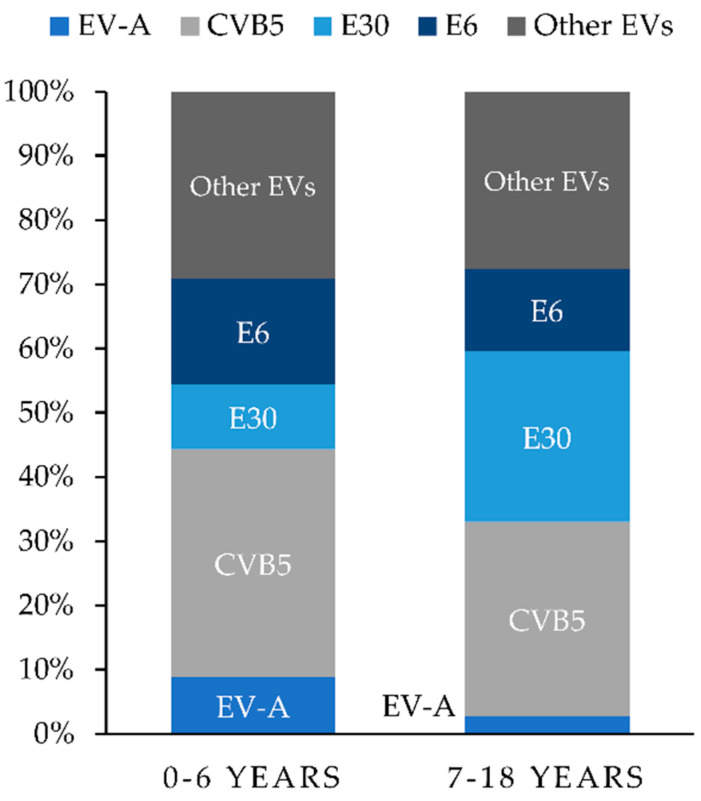
Age distribution by enterovirus types isolated from children with infection of the CNS attributed to enteroviruses in Bialystok, Poland from 2015 to 2019. Abbreviations: EVs, enteroviruses; EV-A, enterovirus A species; CVB5, coxsackievirus B5; E30, echovirus 30; E6, echovirus 6.

The age distribution by the type of enterovirus is shown in Figure 3. Enterovirus species A, CVB5 and E6 were more prevalent in children aged 6 years or younger. E30 was commonly isolated in children over the age of six. The majority (98%) of children were diagnosed with meningitis. Five (2%) of 224 children presented with signs of encephalitis. Virus typing was performed in 2 of those 5 detecting CVB5 in a 3-year-old girl and coxsackie A2 in a 5-year-old boy. No deaths associated with enteroviruses were recorded during the study period.

The most common signs of the infection were headaches and fever, followed by vomiting and neck stiffness. Photophobia was a rarely reported symptom, observed in 42 (19%) children only. Fever was slightly more common and lasted longer in children infected with CVB5 when compared to other EV types. Children with CVB5 also showed lower serum concentrations of CRP and higher numbers of leukocytes in the CSF, in comparison with other types of EV (Table 2). The percentage of lymphocytes in the CSF in children with CVB5 was slightly higher than in other groups. When absolute numbers of lymphocytes in the CSF were compared, the CVB5 group had significantly higher numbers of CSF lymphocytes in comparison to all other groups (*p* < 0.01 for all comparisons). Children with CVB5 were more likely to have negative PCR for enteroviruses in CSF samples as compared to E30. Importantly, the median time from symptoms onset to lumbar puncture was longer in those children, when compared to the E30, but not to other groups.

### 3.3. Sequence Analysis

In order to further characterize the isolates from Poland and examine them in a global context, a phylogenetic analysis was done for the three most frequently detected types. A total of 125 complete VP1 sequences of E30, CVB5 and E6 (E30 = 61, CVB5 = 37 and E6 = 27) were analyzed. All analyzed sequences were submitted to the GenBank sequence database. Closely related sequences available in GenBank and archival Polish sequences were added to the analysis for comparison. International sequences were selected on the basis of their genetic relationships, and archival Polish sequences were selected to represent different clads. We did not analyze recombination events, as for this purpose an analysis of the non-structural genome region is crucial. Phylogenetic trees were constructed applying the neighbor-joining method using the Kimura 2-parameter model in the MEGA program.

#### 3.3.1. E30

In general, nucleotide sequence divergence in pairwise comparisons among Polish E30 isolates ranged from 0.0% to 22.5% (0.0–9.9% *aa* divergence). It depended on the year of detections, varying in 2018 from 0.0% to 21.9% (0.0–7.8% *aa* divergence), and in 2019 from 0.0% to 2.8% (0.0–2.4% *aa* divergence).

Nucleotide sequence analysis has shown that Polish E30 sequences segregated into three distinct major groups. Group 1 represented one isolate from 2017, group 2 comprised three strains from 2018 (0.0–0.1% *nt*; 0.0% *aa* divergence), and group 3 included 33 isolates from the outbreak in 2018–2019 (0.0–3.5% *nt*; 0.0–2.4% *aa* divergence).

**Figure 4:**
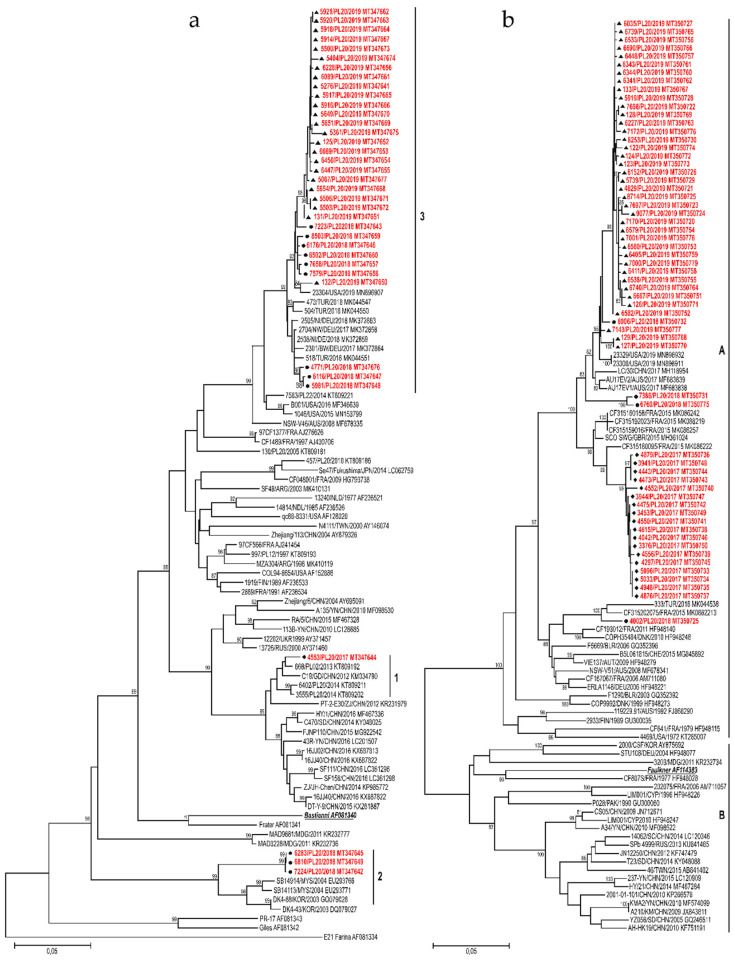

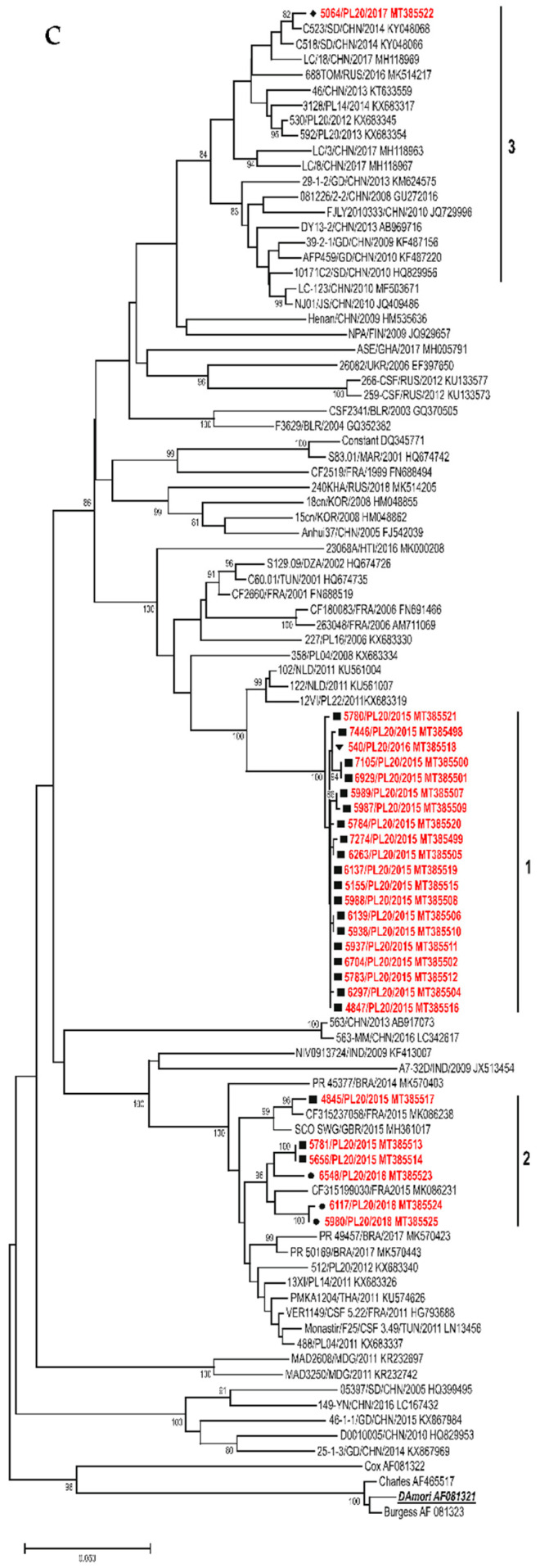
Phylogenetic trees depicting the relationships between a complete VP1 coding region of Polish E30 (**a**), CVB5 (**b**), and E6 strains (**c**) isolated from 2015 to 2019 and sequences from the GenBank. Each strain is referenced by its geographical origin and its accession number. The tree was constructed by the neighbor-joining method and evaluated with 1000 bootstrap pseudoreplicates. Only bootstrap values ≥80% are indicated. In the analyses, genetic distances were calculated with Kimura 2-parameter algorithm. Analyses were conducted in MEGA 10.0.5.

The phylogenetic tree was constructed in order to specify the genetic relationships between the Polish strains and to elucidate the genetic relationship with other strains isolated worldwide in the last decades. Sixty-three sequences used in the analysis were all complete VP1 sequences of E30 available in GenBank. Strains from Poland had the closest genetic relationship with isolates previously identified in European countries (Germany, Turkey, France) but also in other parts of the world (China, USA, Malaysia, South Korea) (Figure 4a). Sequences of outbreak isolates from 2018–2019 grouped together with those from Germany and Turkey from 2017–2018. The one Polish strain from 2017 clustered together with Chinese isolates (2012–2016) and Polish strains (2013–2014) isolated during a previous outbreak [17]. Three strains from 2018 (group 2) had the closest genetic relationship with isolates previously identified in Malaysia and South Korea (2003–2004).

The complete VP1 coding sequence consisted of 295 *aa*; 86.4% (255 of 295) of *aa* sites were conserved between Polish strains. A significant number of *aa* substitutions was observed, with 40 sites of 295 *aa* residues had been changed among the isolates. In most of the polymorphic sites, amino-acid substitutions were associated with the clustering.

#### 3.3.2. CVB5

CVB5 isolates from 2017 to 2019 were genetically homogenous, presenting 0.0% to 10.4% nucleotide divergence (0.0–0.7% *aa* divergence). The genetic diversity varied in 2017 from 0.0% to 1.2% (0.0% *aa* divergence), in 2018 from 0.7% to 10.4% (0.0–0.4% *aa* divergence), and in 2019 from 0.0% to 2.6% (0.0–0.7% *aa* divergence).

Nucleotide sequence analysis showed the Polish CVB5 sequences were classified into genogroup B according to nomenclature proposed by Henquell et al. [18].

The phylogenetic tree was constructed for a total of 110 sequences: 61 Polish and 49 complete VP1 sequences of CVB5 available in GenBank. Strains from Poland had the closest genetic relationship with isolates previously identified in European countries (France, Turkey, UK) but also in other parts of the world (China, USA, Australia) (Figure 4b). Sequences of the 2017 outbreak isolates grouped together with those from France and Great Britain from 2015. Isolates from the outbreak in 2019 clustered together with Chinese, Australian and American isolates from 2017–2019.

The complete VP1 coding sequence consisted of 283 *aa*, with 97.9% (277 of 283) of *aa* sites were conserved between Polish strains. A very limited number of *aa* substitution was observed. Only 6 sites of 283 *aa* residues had been changed among the isolates. In most of the polymorphic sites, amino-acid substitutions were detected in only few strains and substitution pattern had no association with the clustering. Only one site showed the *aa* conservation specific for genetic clustering. It was the I248V substitution found in all isolates from 2017.

#### 3.3.3. E6

Homologous comparison among Polish E6 isolates revealed 0.0–18.9% VP1 nucleotide sequence divergence (0.0–3.8% *aa* divergence). The genetic diversity of Polish E6 varied greatly from year to year, in 2015 ranged from 0.0% to 17.3% (0.0–3.8% *aa* divergence), and in 2018 from 0.2% to 3.1% (0.4–1.0% *aa* divergence).

Nucleotide sequence analysis showed that Polish E6 sequences segregated into three distinct major groups. Group 1 included 20 isolates from the outbreak in 2015–2016 (0.0–0.8% *nt*; 0.0–0.4% *aa* divergence), group 2 comprised 6 strains from 2015–2018 (0.0–5.1% *nt*; 0.0–1.4% *aa* divergence), and group 3 represented one isolate from 2017.

The phylogenetic tree was constructed for 27 Polish strains and 72 complete VP1 sequences of E6 available in GenBank. Strains from Poland had the closest genetic relationship with isolates previously identified in European countries (France, Netherlands, UK, Russia) but also in other parts of the world (China, Brazil) (Figure 4c). The 2015–2016 outbreak isolates (group 1) grouped together with those from the Netherlands from 2011, but also with previously isolated Polish and French strains (2006–2011). One Polish strain from 2017 clustered together with strains isolated during previous outbreaks in Poland (2012–2014) [19], but also with Chinese (2008–2017), and Russian isolates (2016). Six strains from 2015–2018 (group 2) had the closest genetic relationship with isolates previously identified in France, UK, Brazil (2014–2017), and Polish environmental isolates from 2011.

The complete VP1 coding sequence consisted of 289 *aa*, and 95.5% (276 of 289) of *aa* sites were conserved between Polish strains. A very limited number of *aa* substitutions was observed; 13 sites of 289 *aa* residues were changed among the isolates. In most of the polymorphic sites, amino-acid substitutions were associated with the clustering. Eight sites showed the *aa* conservation specific for genetic groups.

### 3.4. Epidemiology and Meteorological Data

**Figure 5:**
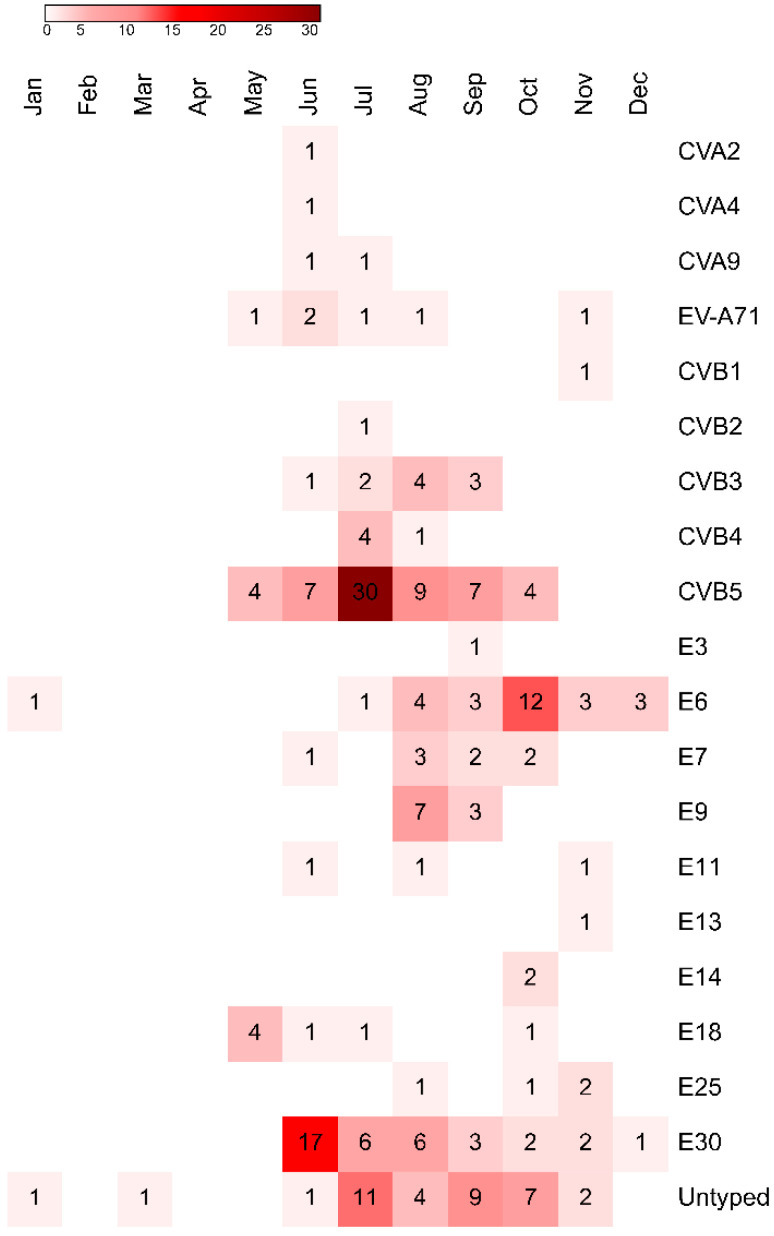
The heatmap showing the temporal distribution of different EV types isolated from samples collected from children, who were hospitalized with aseptic meningitis and encephalitis in Bialystok, Poland from 2015 to 2019. Abbreviations: CV, coxsackievirus; E, echovirus.

The temporal distribution of different EV types is shown in Figure 5. Most cases were reported in the summer season. Number of cases caused by E30 peaked in June, whereas CVB5 peaked in July, and E6 in October.

**Figure 6:**
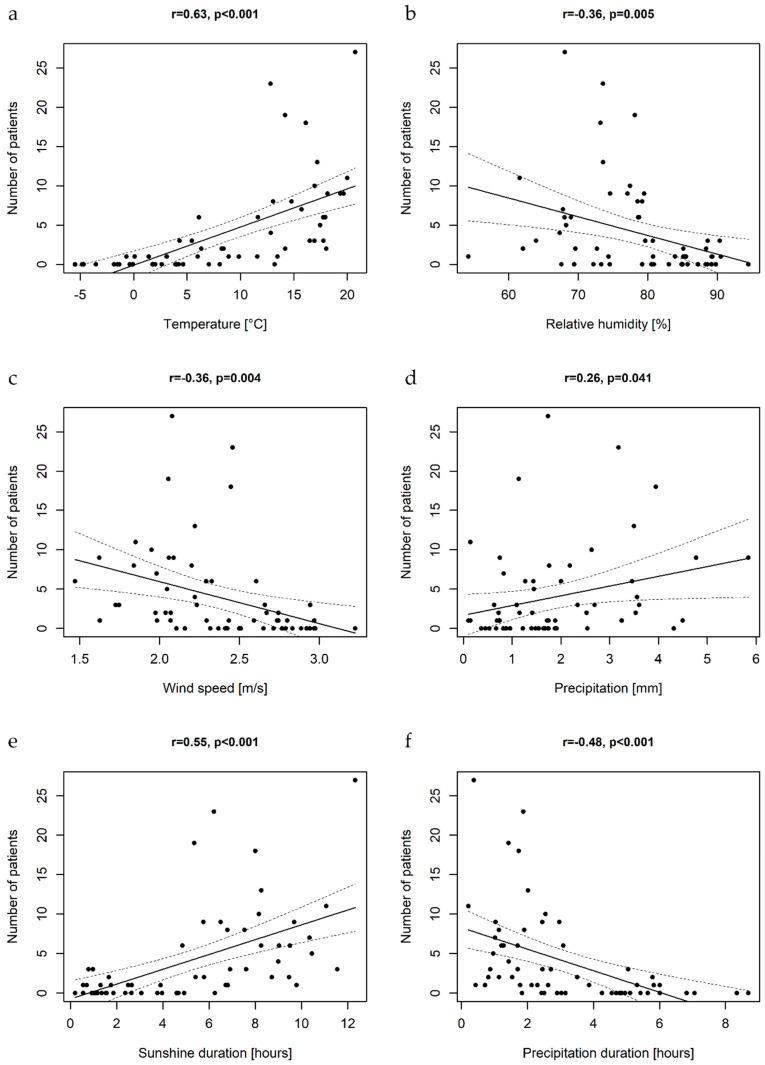
The scatterplots illustrating the associations between mean air temperatures (**a**), relative humidity (**b**), wind speed (**c**), precipitation rates (**d**), sunshine duration (**e**), precipitation duration (**f**), and monthly number of cases of pediatric EV central nervous system infections with the linear regression curves; r-Person’s correlation coefficient.

The analysis of climatic factors revealed that monthly number of cases correlated with mean air temperature, sunshine duration, precipitation (positive association), wind speed, duration of precipitation and relative humidity (negative association) (Figure 6).

In order to select independent climatic factors associated with the incidence of enteroviral disease of the CNS, the GLMM was applied for data analysis. First, the variables were analyzed with univariable GLMM, i.e., the associations between the meteorological factors and the monthly number of EV cases were modeled for each factor separately (Table 3). The univariable models showed that the increase in mean air temperatures, sunshine duration and precipitation rates are associated with the increase in hospitalization rates for enteroviral CNS disease, while the increase in mean wind speed, mean relative humidity and duration of precipitation are associated with the decrease of hospitalization rates.

Then, a model with multiple variables was built. The results of the multivariable analysis are shown in Table 3. Among all analyzed climatic factors, only mean air temperatures and precipitation rates appeared significant in the multivariable model. This indicates that these two environmental factors are independently associated with the number of pediatric enteroviral infections of the CNS in Northeastern Poland. The obtained estimates indicate that in a given month the 1 °C increase in the mean daily temperatures is associated with a 17% (95% CI, 14–20%) increase in the risk of EV infection, while the 1 mm increase in the mean daily precipitation rates is associated with 11% (95% CI, 0–23%) increase in that risk.

The calculated coefficient of determination of the fitted model (R^2^) was 49.4%. This indicates that about 50% of the variation in the number of enteroviral CNS infections can be explained by the variation in the mean air temperatures and precipitation rates.

**Figure 7:**
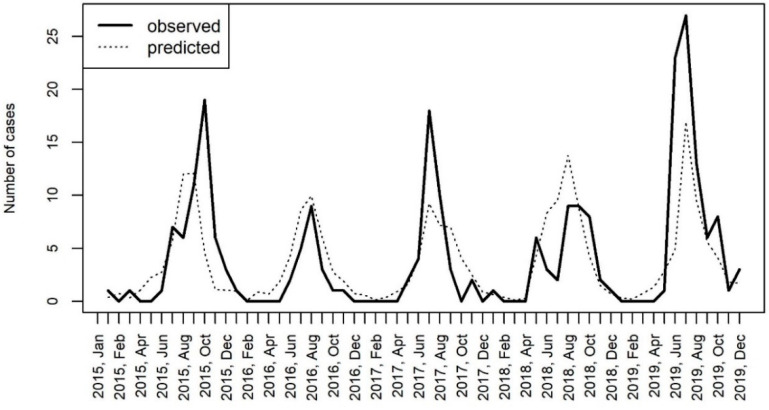
The comparison of hospitalizations for CNS infections caused by enteroviruses in Bialystok, Poland with the number of cases predicted by the generalized linear mixed effects model (GLMM) including the temperature and the precipitation as covariates.

The model with the two variables, the temperature and precipitation, was used for the prediction of hospitalization rates in the considered time period. The comparison of the hospitalization rates that were observed in the study and predicted by the model are shown in Figure 7.

## 4. Discussion

The present study provides an analysis of enteroviruses detected in children hospitalized with infections of the CNS in Northeastern Poland over the period of five years, from 2015 to 2019. Enterovirus surveillance is important because different types can be associated with varying clinical manifestations and outcomes. Changes in predominant types can be accompanied by large-scale outbreaks of enteroviral infections [20]. Here we show that, consistent with other studies, EV-B are the major cause of neurological disease associated with enteroviruses in Northeastern Poland [21]. Enteroviruses belonging to species A were rarely detected. We found that six meningitis cases were caused by endemic circulation of EV-A71, which is an important global infectious disease threat due to its potential to cause outbreaks and severe neurologic disease [22]. Since 2014, a growing number of enterovirus D68 outbreaks associated with severe respiratory diseases and neurological complications have been occurring in different countries. Enterovirus D68 has also been detected in children with aseptic meningitis [23]. We did not use primers to detect VP1 sequences of EV-D species. However, viral culture and typing successfully identified a viral type in 188 of 195 children implying that, even if missed, EV-D are not a common cause of meningitis in Poland.

Coxsackievirus B5 was the most frequently detected enterovirus type, followed by E30 and E6. All three types showed epidemic patterns with outbreaks in 2015 (E6), 2017 (CVB5) and 2019 (CVB5 and E30). Outbreaks of meningitis associated with E30 [21,24,25,26,27] and E6 [28,29,30,31] were frequently reported in many parts of the world. Similarly, outbreaks of CVB5 meningitis were reported previously [32,33,34]. However, according to a recent review, CVB5 is implicated in only a small proportion of neurological disease caused by enteroviruses globally [6].

Population immunity to a particular EV type determines the potential extent of the virus spread. Periodic increases in levels of EV circulation are probably caused by accumulation of a susceptible population during the years of virus quiescence [2]. However, sharp increases in the number of enteroviral infections might also be caused by the emergence of an immune escape mutant. Enteroviruses are among the most rapidly changing RNA viruses. Viral RNA polymerases frequently introduce substitutions to the synthesized genome. Consequently, enterovirus populations exist as quasispecies, or collections of closely related viral genomes that differ by only one or a few substitutions [20]. Existence of a swarm of viral variants has been shown to determine neuropathology and immune evasion through cooperative interactions in a viral population [35]. The volume of global travel is expanding exponentially. The increased number of travelers allows mixing of microorganisms from different regions, resulting in an increase in their genetic variability [36]. Enteroviruses identified in our study were related to other strains previously detected in many other parts of the world. The majority of the E30 outbreak isolates from 2018–2019 clustered together with strains isolated 1–2 years before in Germany and Turkey, which can be explained by travel preferences of people in Poland. According to the Polish Ministry of Sport and Tourism, Germany was the most popular destination for Polish travelers in recent years [37]. Similarly, the 2017 outbreak of CVB5 and 2015 outbreak of E6 infections could be associated with travel to the UK, France or the Netherlands, which are also visited by Polish travelers frequently. There was, however, an interesting difference in temporal patterns of those viruses. The emergence of CVB5 strains in Poland in 2017 was preceded by the detection of closely related viruses in Europe just two years before. Echovirus 6 on the other hand was circulating in Europe from 2006, before causing an outbreak in Bialystok, Poland in 2015.

VP1 genotyping by phylogenetic analysis can differentiate lineages within a particular type in order to identify emerging variants or types. Isolates of E30 and E6 had a surprisingly high genetic diversity in this study. VP1 sequences corresponded closely with those obtained from many parts of the world, suggesting co-circulation of multiple transmission chains. This provides a reservoir from which novel variants may potentially emerge, perhaps as another outbreak event. In fact, waves of echovirus 30 activity were reported to be caused by new genomic lineages, which replaced previously circulating ones [2]. In contrast to E30 and E6, CVB5 isolates had low genetic diversity in this study. Still, CVB5 was the most commonly isolated EV type. Coxsackievirus B5 isolates from the 2019 outbreak were related to isolates from China, Australia and the USA from 2017–2019. Those countries are not among the top travel destinations of Polish travelers. This huge geographical distance and a short time interval between detection of closely related strains in Poland, China, Australia and the USA indicate that CVB5 outbreaks can originate from a new genetic strain introduced from a distant part of the world. Frequent travel might increase importation of other CVB5 lineages resulting in another outbreak. Previously, increased activity of CVB5 has been associated with the emergence of new genetic lineages with high levels of nucleotide identity between isolates from the same outbreak [38].

Animal models and clinical observations have revealed different host immune responses to infections with different enteroviruses [39]. In this study infections caused by CVB5 were characterized by more common and longer duration of fever, higher CSF pleocytosis secondary to lymphocytosis and lower CRP concentrations, when compared to other EV types. High levels of white blood cells in the CSF with a predominance of lymphocytes were detected previously in patients with fatal or severe infections caused by EV-A71 [40,41]. It was hypothesized that lymphocytes play a central role in the immunopathology of those infections. We have previously shown that in children with enteroviral meningitis, lymphocytes in the CSF correlate with CSF concentrations of total tau protein, which is a marker of brain parenchymal damage [42]. However, the detrimental role of lymphocytes has been questioned, as these lymphocytes now are suggested to be protective. In animal models, lymphocytes in the CNS function to reduce the mortality and tissue viral loads [43]. Moreover, intravenous immunoglobulins, which are produced by B lymphocytes, have been shown to be effective in treatment for patients with neurological symptoms infected with enteroviruses [44,45]. The importance of humoral immune responses in clearing enteroviral infection was also demonstrated by the chronic or life-threatening disease that is observed in patients with agammaglobulinemia [46]. High CSF lymphocytosis concurrent with longer duration of fever in children infected with CVB5 in our study might be a reflection of higher virulence of this EV type in comparison to other EVs. However, we found slight differences in the time interval from symptoms onset to lumbar puncture between the groups that could have affected those observations. It has been shown previously that neutrophils in the CSF predominate in the early phase of viral meningitis, with lymphocyte influx occurring over the following 6–48 h [47]. Nevertheless, our data confirm the notion that non-polio EVs should not be considered as a homogenous group, but rather a collection of viral agents having differing degrees of tissue tropism and virulence.

Enteroviruses were detected year-round in this study. Consistent with the seasonality of EV infections in temperate climates, we have shown that EV-associated infections of the CNS were more prevalent during the summer-fall season [26,48,49,50,51]. We have also noted small differences in the peak months by different EV types. Infections caused by E30 peaked early in June, CVB5 in July, whereas E6 late in October. The causes of enterovirus types circulating earlier than others remains unclear. Pons-Salort et al. [3] hypothesized that a later peak of historical poliomyelitis when compared with the peak of enterovirus cases was caused by the longer survival of the virus in the environment than in droplets or aerosols. That allowed for longer periods of fecal-oral transmission compared with the respiratory route. The explanation for the later peak month of E6 observed in our study could be that fecal-oral transmission for the E6 accounts for more transmission compared with E30 and CVB5, in which respiratory routes possibly play a relatively larger role.

We attempted to identify drivers of EV seasonality by analyzing climatic factors and monthly numbers of hospitalizations for all-type EV infections of the CNS. The Pearson’s correlation coefficient revealed that multiple climatic factors correlate with the number of cases. In the analysis of grouped data in the mixed-effect model, mean air temperatures and precipitation rates were the two climatic variables that explained nearly 50% of the variation in the number of hospitalizations. The recent analysis of spaciotemporal distribution of enteroviral disease in the USA gave important insights into drivers of enterovirus epidemiology. Intensity of enterovirus transmission was found to be affected by the dew point temperature, which is a measure of humidity [3]. Since enteroviruses can be transmitted by droplets, virus survival in aerosols is important. According to laboratory experiments, it can be positively affected by relative humidity [52]. We did not find independent relations with relative humidity, but we found positive associations with precipitation rates, which have an impact on humidity.

There are several limitations to this study. First, stool samples are best for EVs detection and seem to be a reliable alternative to CSF testing in CNS infections caused by enteroviruses [53]. However, the presence of virus in these samples was questioned as an evidence of etiology because viral shedding is common and may occur asymptomatically [54]. Second, in the study we enrolled children from Northeastern Poland only. These results might not be representative of enterovirus circulation in the entire Poland. Third, we analyzed climatic factors recorded in Bialystok only, whereas some of the children recruited in the study lived outside Bialystok and could have been affected by slightly different weather conditions. Nevertheless, this is the first study describing molecular epidemiology of infections of the CNS caused by enteroviruses in Polish children.

## 5. Conclusions

This study demonstrates that CVB5, E30 and E6 are predominant enterovirus types in children with infections of the CNS in Northeastern Poland. Phylogenetic analyses of these three EV types show multiple lineages co-circulating in this region. From this swarm, more pathogenic or neurotropic variants may emerge, perhaps as another outbreak event. Outbreaks can also originate from a new virus type introduced from a distant part of the world. The potential extent of the virus spread is affected not only by the population immunity, but also by climatic factors, of which mean air temperatures and precipitation rates were of large importance in this study. Continued monitoring and surveillance of enteroviral infections of the CNS are therefore imperative.

## Figures and Tables

**Table 1:** Enterovirus types identified between 2015 and 2019 in children with infection of the CNS attributed to enteroviruses in Bialystok, Poland divided by year.

		EV-A				EV-B														
**Year**	EVs Typed n (%)	CVA2	CVA4	CVA9	EV-A71	CVB1	CVB2	CVB3	CVB4	CVB5	E3	E6	E7	E9	E11	E13	E14	E18	E25	E30
**2015**	28/54 (52%)					1			2			22			2			1		
**2016**	14/22 (64%)				1			1				1	2	8				1		
**2017**	33/35 (94%)			1	1		1	4	2	18	1	1		2					1	1
**2018**	38/38 (100%)				2					4		3	6		1		2	5	3	12
**2019**	75/75 (100%)	1	1	1	2			5	1	39						1				24
**Total**	188/224 (84%)	1	1	2	6	1	1	10	5	61	1	27	8	10	3	1	2	7	4	37

Abbreviations: EVs, enteroviruses; EV-A, enterovirus A species; EV-B, enterovirus B species; CV, coxsackievirus; E, echovirus.

**Table 2:** Clinical features of the study population by type of enteroviruses.

	Enteroviruses—All	Coxsackie B5	Echovirus 30	Echovirus 6	Other EV Types
Sex					
Female; ***n* (%)**	77 (100%)	21 (27%)	14 (18%)	12 (16%)	30 (39%)
Male; ***n* (%)**	147 (100%)	40/61 (27%)	23 (16%)	15 (10%)	69 (47%)
Male-to-female ratio	1.91	1.90	1.64	1.25	2.3
Age **(years)**	8.1 (5.4–12.7)	8.6 (5.7–12.8)	12.6 (8.3–15.1) ^4^	7.2 (5.4–13.7)	7.5 (5.0–11.0)
Age groups					
<1; ***n* (%)**	3 (100%)	0 (0%)	0 (0%)	0 (0%)	3 (100%)
1–3; ***n* (%)**	18 (100%)	6 (33%)	0 (0%)	1 (6%)	11 (61%)
4–6; ***n* (%)**	74 (100%)	22 (30%)	8 (11%)	12 (16%)	32 (43%)
7–13; ***n* (%)**	89 (100%)	19 (21%)	16 (18%)	9 (10%)	45 (51%)
14–17; ***n* (%)**	40 (100%)	14 (35%)	13 (33%)	5 (12%)	8 (20%)
Clinical presentation					
Meningitis; ***n* (%)**	219 (100%)	60 (27%)	37 (17%)	27 (12%)	95 (44%)
Encephalitis; ***n* (%)**	5 (2%)	1 (20%)	0 (0%)	0 (0%)	4 (80%)
Symptoms onset to CSF collection **(days)**	2 (1–4)	2 (1–4) ^2^	1 (1–2) ^1,3,4^	2 (1–6) ^2^	2 (1–4) ^2^
Length of hospital stay **(days)**	7 (7–9)	8 (7–10) ^2^	7 (6–7) ^1,4^	7 (6–10)	7 (7–9) ^2^
Only stool PCR positive; ***n* (%)**	40/224 (18%)	21/61 (34%) ^2^	0/37 (0%) ^1^	6/27 (22%)	13/99 (13%)
Signs and symptoms					
Headaches; ***n* (%)**	221/224 (99%)	59/61 (97%)	37/37 (100%)	27/27 (100%)	98/99 (99%)
Headaches **(days)**	2.5 (2–4)	3 (2–5)	2 (2–3)	2 (1–3)	2 (2–4)
Fever; ***n* (%)**	203/224 (91%)	61/61 (100%)	29/37 (85%)	23/27 (85%)	90/99 (91%)
Fever **(days)**	2 (1–3)	3 (1–5) ^2,3,4^	1 (1–2) ^1^	1 (1–2) ^1^	2 (1–2) ^1^
Vomiting; ***n* (%)**	170/224 (76%)	47/61 (77%)	28/37 (76%)	19/27 (70%)	76/99 (77%)
Vomiting **(days)**	1 (1–1)	1 (1–2)	1 (1–2)	1 (0–1)	1 (1–1)
Photophobia; ***n* (%)**	42/224 (19%)	5/61 (8%)	9/37 (24%)	7/27 (26%)	21/99 (21%)
Neck stiffness; ***n* (%)**	164/224 (73%)	37/61 (61%)	24/37 (65%)	21/27 (78%)	82/99 (83%)
Tremor; ***n* (%)**	3/224 (1%)	1/61 (2%)	0/37 (0%)	0/27 (0%)	2/99 (2%)
Seizures; ***n* (%)**	2/224 (<1%)	1/61 (2%)	0/37 (0%)	0/27 (0%)	1/99 (1%)
Altered level of consciousness; ***n* (%)**	8/224 (4%)	1/61 (2%)	0/37 (0%)	2/27 (7%)	5/99 (5%)
CRP **(mg/L)**	3.0 (1.0–11.0)	1.2 (0.5–2.9) ^2,3,4^	7.7 (2.2–16.0) ^1^	6.0 (1.5–12.5) ^1^	5.2 (2.5–12.7) ^1^
CRP > 10 mg/L; ***n* (%)**	57/223 (26%)	5/61 (8%) ^2^	15/36 (42%) ^1^	7/27 (26%)	30/99 (30%)
ALT **(IU/mL)**	11 (9–14)	10 (9–13) ^4^	11 (9–14)	12 (10–14)	12 (10–14.5) ^1^
AST **(IU/mL)**	22 (17–26)	20 (16–24) ^4^	20 (14–25) ^4^	25 (19–28)	23.5 (19–27) ^1,2^
WBC **(×10^9^ cells/L)**	8.8 (6.9–11.1)	8.2 (6.9–11.0)	8.7 (6.3–10.9)	10.3 (7.9–13.3)	8.7 (7.0–11.5)
Blood lymphocytes **(%)**	28 (18–37)	33 (25–43) ^3^	26 (18–31)	16 (12–21) ^1,4^	27 (20–40) ^4^
Blood neutrophils **(%)**	61 (51–73)	55 (45–63) ^3^	62 (56–71)	78 (69–82) ^1,4^	60 (47–73) ^3^
Blood monocytes **(%)**	9 (7–12)	9 (8–11)	9 (7–12)	8 (6–9)	10 (7–12)
CSF protein **(g/L)**	0.33 (0.25–0.44)	0.39 (0.30–0.55) ^4^	0.31 (0.24–0.38)	0.31 (0.28–0.40)	0.29 (0.24–0.43) ^1^
CSF cells **(/µL)**	137 (47–364)	249 (136–618) ^2,3,4^	73 (37–190) ^1^	124 (48–170) ^1^	86 (26–290) ^1^
CSF lymphocytes **(%)**	60 (32–80)	73 (48–83) ^2^	46 (30–68) ^1^	55 (21–68)	55 (30–80)
CSF neutrophils **(%)**	26 (9–57)	16 (6–43) ^3^	26 (14–64)	35 (22–76) ^1^	29 (24–43)
CSF monocytes **(%)**	7 (2–13)	7 (3–13)	9 (5–15)	4 (1–10)	7 (2–12)
CSF neutrophils >50%; ***n* (%)**	62/203 (31%)	11/58 (29%)	12/34 (35%)	10/24 (42%)	29/87 (33%)

Abbreviations: WBC, White blood cell count; CSF, cerebrospinal fluid; ALT, alanine aminotransferase; AST, aspartate aminotransferase; *p* < 0.05: ^1^ vs. CVB5; ^2^ vs. E30; ^3^ vs. E6; ^4^ vs. other EV types.

**Table 3:** Predictive factors of the number of cases of pediatric enteroviral infections of the CNS by uni- and multivariable generalized linear mixed models (GLMM). The coefficient of determination (R^2^) of the multivariable model was 49.4%.

	Univariable Analysis		Multivariable Analysis	
	RR (95% CI)	*p*	RR (95% CI)	*p*
**Temperature (°C)**	1.17 (1.15–1.2)	<0.001	1.17 (1.14–1.2)	<0.001
**Sunshine duration (hours)**	1.26 (1.21–1.31)	<0.001		
**Wind speed (m/s]**	0.25 (0.18–0.34)	<0.001		
**Relative humidity (%)**	0.95 (0.94–0.96)	<0.001		
**Precipitation (mm)**	1.45 (1.32–1.6)	<0.001	1.11 (1.00–1.23)	0.043
**Precipitation duration (hours)**	0.58 (0.52–0.65)	<0.001		

Abbreviations: RR, Relative Risk; 95%CI, 95% confidence interval.
